# Stronger Lewis Base Antisolvents Improve Perovskite Nanocrystal Stability

**DOI:** 10.1021/acsenergylett.6c00480

**Published:** 2026-04-09

**Authors:** Junzhi Ye, Charlie Nicholls, Woo Hyeon Jeong, Dong Yoon Chung, Ashish Gaurav, Kieran De-Ville, Rui Xu, Zongming Ni, Qingyu Wang, Xinyu Shen, Jieling Tan, Eilidh L. Quinn, Maxime Atkinson, Wei Zhang, Haitao Zhao, Henry J. Snaith, Robert A. Taylor, Yunwei Zhang, Robert L. Z. Hoye

**Affiliations:** † Department of Chemistry, Inorganic Chemistry Laboratory, 150602 University of Oxford, Oxford, OX1 3QR, United Kingdom; ‡ Institute of Polymer Optoelectronic Materials and Devices, Guangdong Basic Research Centre of Excellence for Energy & Information Polymer Materials, State Key Laboratory of Luminescent Materials and Devices, School of Materials Science and Engineering, 26467 South China University of Technology, Guangzhou 510640, China; § School of Physics, 626014 Sun Yat-sen University, Guangzhou, 510275, China; ∥ 47892 Wenzhou Institute of Technology, Wenzhou, 325000, China; ⊥ Clarendon Laboratory, Department of Physics, 6396 University of Oxford, Oxford, OX1 3PU, United Kingdom; ○ Center for Alloy Innovation and Design (CAID), State Key Laboratory for Mechanical Behavior of Materials, 12480 Xi’an Jiaotong University, Xi’an, 710049, China; □ Research Centre for Materials Intelligent Manufacturing, State Key Laboratory of Ultra-precision Machining Technology, Department of Electrical and Electronic Engineering, 26680 The Hong Kong Polytechnic University, Hong Kong, China

## Abstract

Lead-halide perovskite nanocrystals (NCs) have gained attention for optoelectronics, but careful selection of the antisolvent used for purification is essential to achieve high monodispersity and yield while minimizing surface damage. Current understanding indicates that this requires lowering the relative polarity of the antisolvent, yet high-polarity antisolvents are widely used for purification, as we confirm through data mining. We show that polarity alone is insufficient for antisolvent selection by comparing ethyl acetate and acetonitrile for CsPbI_3_ NC purification. Despite its higher polarity, acetonitrile yields improved colloidal stability compared to ethyl acetate. Using ^1^H NMR, FTIR, and XPS measurements, alongside DFT calculations, we demonstrate that acetonitrile acts as a stronger Lewis base, binding to and passivating the NC surface. Coordination of acetonitrile to the perovskite NC surface enhances stability and improves their performance in light-emitting diodes. These findings establish a mechanistic framework for antisolvent selection to realize bright and stable halide perovskite NCs.

## Graphical abstract

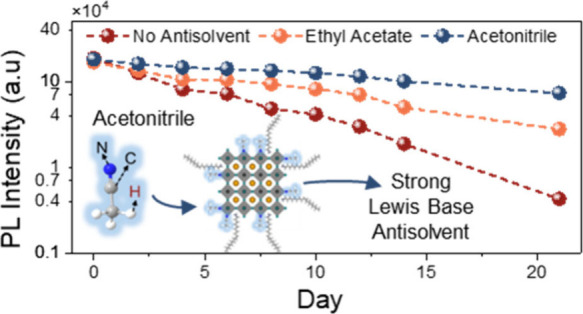

Colloidal lead-halide perovskite nanocrystals (NCs) have rapidly risen in prominence as an emerging class of solution processable semiconductors capable of achieving sharp emission with high photoluminescence quantum yields (PLQYs). This makes them promising for a wide range of optoelectronic applications, including light-emitting diodes (LEDs), photovoltaics, lasers, and single-photon emitters.^1–9^ For all applications, it is critical that the NCs are monodisperse, with unreacted precursors and other impurities removed. This is achieved through purification, where as-synthesized colloidal NCs are mixed with polar antisolvents, and centrifuged to separate the desired product from the crude solution.^10^ However, this step inevitably introduces defects to the NC surface when the labile ligands are removed. Surface damage is detrimental to both the optoelectronic properties and stability of these NCs.^11–14^ It is possible to precipitate out NCs without adding an antisolvent when sufficient sedimentation velocity is reached during centrifugation, but this can give rise to lower yields, with fewer impurities removed.^15–17^ Other purification methods, such as size-exclusion chromatography and cross-flow membrane filtration, can purify these materials without changing solvent polarity, but they are typically slower and can be more challenging to scale cost-effectively.^18^ In contrast, purification through the addition of polar antisolvents can produce high yields and are scalable, but understanding the design principles for the most effective antisolvents that minimize damage to the NCs is critical.

Although there has been progress in improving the colloidal stability of Br-based NCs,^19^ I-based NCs (especially CsPbI_3_) still have poor colloidal stability under ambient conditions. For example, the PLQY of colloidal CsPbI_3_ NCs has been reported to decrease from >60% to less than 1% after a day in ambient air.^20,21^ So far, most groups have focused on identifying passivation strategies to compensate for ligand and halide loss during purification.^8,14,22–26^ In our previous work, we found that surface damage to the NCs could be reduced by using lower polarity antisolvents.^11^ In a system where the NCs are coordinated with oleate and oleylammonium species (i.e., X-type ligands), increasing the relative polarity of the antisolvent (e.g., by using acetone or butanol instead of ethyl acetate) results in greater ligand removal because of ligand detachment induced through proton transfer (if protic antisolvents are used),^14^ or by encouraging amide formation via condensation reactions between the two ligands (occurs regardless of whether the antisolvent is protic).^11^ We found that I is particularly severely affected, such that a mixed I–Br system would have the I selectively etched away. We proposed that this is because the I is bound to the ammonium groups in oleylammonium through H bonding, as well as the Pb–I bond being weaker than the Pb–Br bond.^11^ There is also a reduction in PLQY and lower stability of the NCs in ambient air when using more polar antisolvents because of the higher surface defect density and reduced concentration of ligands on the surface of the NCs.^11,27–30^ This implies that low-polarity antisolvents (e.g., methyl acetate or ethyl acetate) should give rise to more stable NCs with higher PLQYs. Despite this, there are many reports of perovskite NCs with improved stability prepared using acetone, high-polarity alcohols or acetonitrile as the antisolvent.^31,32^

To quantify this, we used data mining^33,34^ through 3109 papers to determine the frequency with which groups have used antisolvents of different polarities (Figure S1, SI). From this, we found that there are more papers reporting the use of ethyl acetate than methyl acetate. This aligns with expectations,^11^ since ethyl acetate has a lower relative polarity (0.228 for ethyl acetate vs 0.253 for methyl acetate). On the other hand, acetonitrile has a higher relative polarity (0.46) and, yet, is more commonly used than ethyl acetate (Figure S1, SI). This suggests that antisolvent polarity is not the only factor influencing NC properties. It is therefore essential to develop improved understanding of how these antisolvents influence the surface chemistry of the NCs, and develop broader, more precise design principles for antisolvent selection.

To systematically gain greater mechanistic insights into the role of the antisolvent during purification, we focused on CsPbI_3_ NCs. We compared purification without the use of antisolvents, and with ethyl acetate or acetonitrile. We selected these two antisolvents for comparison because they are a pair that clearly deviates from expectations, in that acetonitrile should have yielded lower PLQYs than ethyl acetate, and yet this is not what is found in the literature. To understand how these antisolvents influence the surface chemistry of CsPbI_3_ NCs, we used proton Nuclear Magnetic Resonance (^1^H NMR), Fourier Transform Infrared Spectroscopy (FTIR) and X-ray Photoemission Spectroscopy (XPS) to determine changes to the density and chemical species of ligands on the surface of the NCs, as well as the nature of surface species on the NCs. We correlated these experiments with first-principles computations using density functional theory (DFT) to understand the nature of how these antisolvents interact with the NC surface. We linked together these new insights into the surface chemistry with changes in the optical properties and stability of the perovskite NCs. We found that there was a difference between the fresh and aged samples. Among freshly purified CsPbI_3_ NCs, higher PLQYs and longer PL lifetimes were obtained from NCs purified with the lower-polarity ethyl acetate. By contrast, acetonitrile-washed NCs showed higher colloidal stability over time under ambient conditions, despite more oleylammonium and oleate ligands being removed initially. This is due to acetonitrile being able to bind to the NC surface through the N group acting as a Lewis base to Pb^2+^ Lewis acid species on the surface, which we confirmed through DFT calculations and NMR, FTIR and XPS measurements. We demonstrate the impact of washing with stronger Lewis base antisolvents on perovskite light-emitting diode (LED) performance. Our findings bring forth a new design principle for selecting antisolvents that goes beyond relative polarity as the sole criterion. That is, selecting ligands with the ability to bind to the NC surface could lead to more stable NCs, as well as more efficient LEDs.

CsPbI_3_ NCs were prepared by our previously reported modified ligand-assisted reprecipitation (LARP) method,^17^ and compared against a hot injection (HI) method.^3^ In both cases, purification was obtained by centrifugation through a two-step process. First, the solutions obtained after synthesis by LARP or HI were centrifuged at 3000 rpm for 3 min. The supernatant obtained was subsequently centrifuged at 12 000 rpm for 5 min, either with no antisolvent added, or with a polar antisolvent added. The precipitate obtained from this second centrifugation step was redispersed in 1 mL hexane and filtered (details in SI).

Herein, we first examined the NCs prepared by LARP. Figure 1(a) shows the steady state absorption and PL spectra of CsPbI_3_ NCs solution purified without using any antisolvent, compared to NCs purified using ethyl acetate or acetonitrile. From Elliott model fitting to the optical absorption edges,^35^ we found that the exciton binding energies were approximately 20 meV for all NCs regardless of the purification process, indicating negligible effect on the dielectric environment due to the change in surface ligands (Figure S2, SI). The PL spectra of the NCs purified without antisolvents (pristine) show a clear dual peak at 640 and 680 nm wavelength (Figure S3­(a), SI), indicating that there is a wider size distribution. Similar phenomena were also observed for NCs prepared by the HI method, as shown in Figure S3­(d),(e). NCs purified with antisolvents exhibited narrower PL peaks centered at 681 nm wavelength, with the fwhm reduced from >41 to 38 nm (Figure S3­(b),(c), SI), and there was no significant difference in the absorption and PL spectra when using ethyl acetate or acetonitrile. This suggests that the antisolvents removed unreacted precursors and narrowed the size distribution of the NCs without changing their mean size or phase purity.

**Figure 1:**
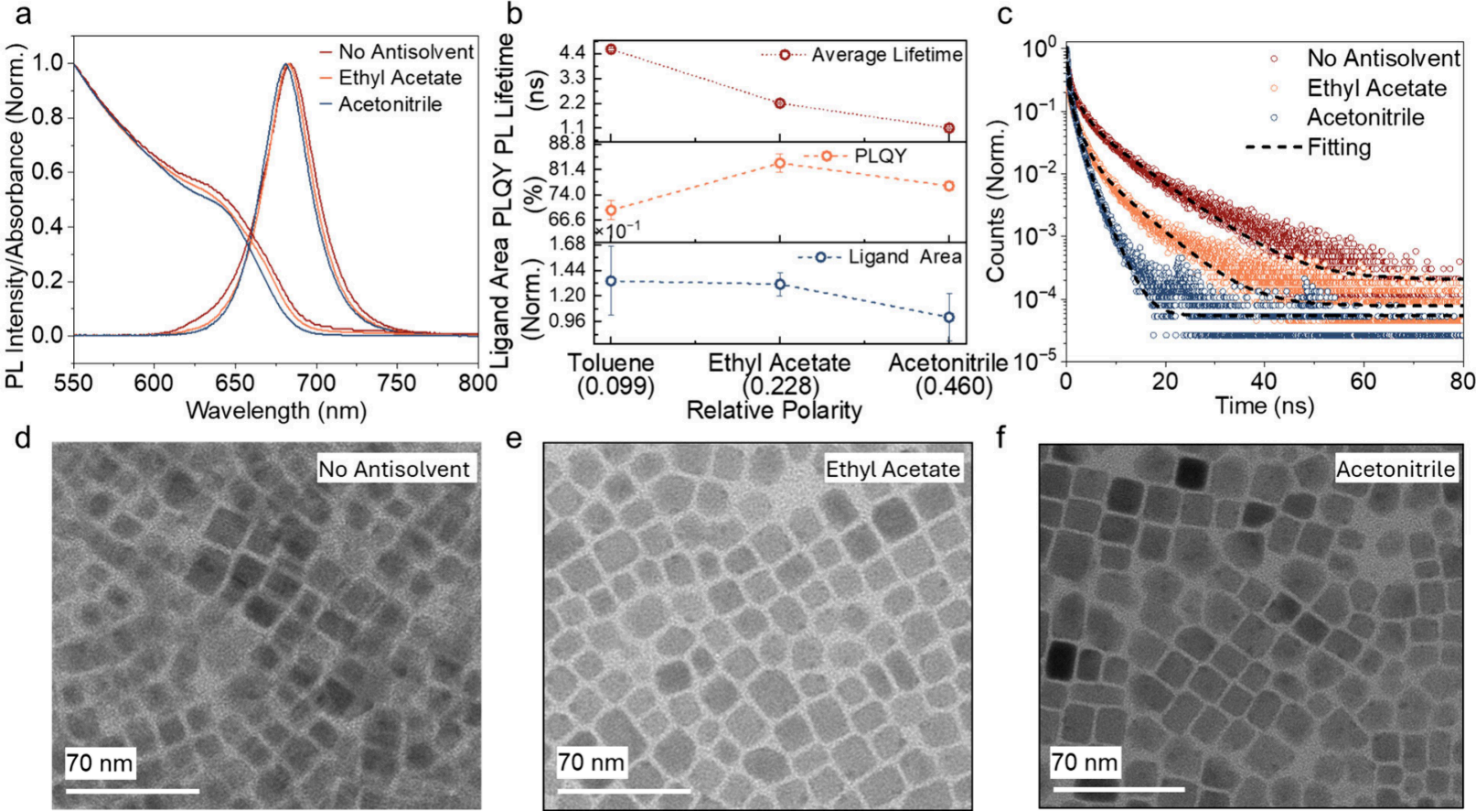
Optical properties of LARP-synthesized CsPbI_3_ NCs after purification. (a) Steady state absorption and photoluminescence (PL) spectra of the NCs in colloidal solution. (b) Change in PL lifetime, PLQY and amount of ligands recovered in the NCs after purification with no antisolvent (i.e., using just the original solvent, toluene), or with ethyl acetate or acetonitrile as the antisolvent. The PL lifetimes were extracted from the data in panel (c), and the error bars represent the uncertainties from fitting the phenomenological triexponential model to the PL decay data (fits shown in panel c; details in Table S2, SI). The PLQY was measured with colloidal NC solutions in 1 mm thick quartz cuvettes using 400 nm wavelength cw excitation laser, with approximately 74.61 mW cm^–2^ power density. The ligand area was quantified based on ^1^H NMR of precipitated NCs redispersed in deuterated toluene. The internal standard was the residual nondeuterated toluene (chemical shift of 7.09 ppm). The error bars for PLQY and ligand area represent sample-to-sample variation and were obtained by repeating the PLQY and NMR measurements for three different batches of NC samples. The dashed lines are added to aid in comparing differences in values across the samples and are not to imply any model fit. (c) Time-resolved PL of NCs purified without using any antisolvent, and with ethyl acetate or acetonitrile as the antisolvent. The PL lifetime was measured from colloidal NCs drop cast onto Si substrates. The measurements were made using confocal PL microscopy with a 400 nm wavelength pulsed pump laser at 418.8 μJ cm^–2^ fluence. (d–f) Transmission electron micrographs of CsPbI_3_ NCs purified without using antisolvents or using ethyl acetate/acetonitrile. Size distribution shown in Figure S5, SI.

Looking in more detail at the LARP-NCs, those prepared with either ethyl acetate or acetonitrile exhibited the same X-ray diffraction (XRD) pattern (Figure S4, SI). Transmission electron microscopy (TEM) images showed the median sizes to be the same (Figure 1(d–f) and Figure S5, SI). However, the antisolvents influenced the optoelectronic properties of the NCs. First, the use of antisolvents slightly decreased the PLQY of the NC solutions, and the more polar acetonitrile antisolvents led to a slightly greater reduction in PLQY (76.6 ± 1.4%) than ethyl acetate (83.3 ± 2.7%). The decrease in PLQY between NCs treated with ethyl acetate and acetonitrile can be explained by the amount of surface ligands removed during purification, as shown in Figure 1(b). We quantified the ligand density on the perovskite NCs using ^1^H NMR (Figure S7, SI) by (1) ensuring that the colloidal NC solutions we were comparing had similar concentrations based on the yields (Table S1 and absorbance at exitonic position (Figure S2, SI), and (2) using residual nondeuterated toluene in the deuterated-toluene solvent as the internal reference peak.^13^ The vinyl peak occurs at a chemical shift of approximately 5.5 ppm, which arises due to the C=C group in the oleylammonium and oleate ligands. The integrated peak area is proportional to the total amount of ligands recovered with the NCs after precipitation.^11,13,36^ By comparing these integrated peak areas (Figure 1(b)), it can be seen that using more polar antisolvents results in greater ligand removal, as expected from our previous findings.^11^ This is consistent with our prior observations that more polar antisolvents leads to the introduction of more surface defects, which reduce the PLQY of the CsPbI_3_ NCs slightly despite its defect tolerance.^11,12^ The deviation from these findings is the NCs purified without antisolvents (i.e., which just had the original toluene solvent). Here, we found that although there was a higher ligand density (from ^1^H NMR) and slower PL decay (Figure 1(b) and (c)), the PLQY is lower (69.5% ± 2.8%). This is due to the removal of fewer impurities, such as excess ligands and precursors, when no antisolvent is used, despite there being less damage to the NC surface.^13,14,37^

As the PLQY was measured from NCs in colloidal solution, the relative change was small due to the high tolerance to defects in I-based NCs.^12^ We therefore measured the PL lifetime of the drop-cast NC films on Si substrates to further investigate the effect of the antisolvents on the initial surface damage to the NCs. This is because NCs in drop-cast films tend to have more defects introduced than in colloidal solution. We extracted time constants from the PL decay curves by fitting a phenomenological triexponential model (Table S2, SI). This model is not physically relevant, but allows us to quantitatively compare the PL decays of the NCs without having to make assumptions about the recombination processes taking place. We found that there was an obvious decrease in the weighted average time constants of the PL decays from 4.6 to 2.2 ns and 0.82 ns as the antisolvent relative polarity increased from 0.099 (toluene; i.e., purified without adding polar antisolvents) to 0.228 (ethyl acetate) and 0.460 (acetonitrile), as shown in Figure 1(b) and (c). The films were measured at a fluence of 418.8 μJ cm^–2^, with a pulsed 400 nm wavelength pump laser. As we tuned the fluence over an order of magnitude from 41.9 μJ cm^–2^ to 418.8 μJ cm^–2^ (Figure S6­(a–c), SI), there was a general decrease in PL time constants with increasing fluence for the NCs purified without antisolvents. By contrast, the NCs purified with antisolvents had a slight increase then decrease in lifetime as the fluence was increased (Figure S6­(d), SI). The latter behavior is consistent with these free-carrier systems changing from a trap-dominated nonradiative recombination regime^38^ to an Auger-dominated recombination regime,^39^ since defects were introduced when using polar antisolvents during purification.^11,12^ However, changes to the PL lifetime with changes in fluence were less pronounced in the samples purified with acetonitrile (Figure S6, SI). This is consistent with a higher defect density being present in the NCs treated with more polar antisolvents, such that a higher fluence is needed to fill these traps before transitioning from a trap-assisted monomolecular to an Auger recombination regime.

Based on these findings, and the current understanding in the literature, we would have expected the acetonitrile-washed NCs to have the lowest stability under ambient conditions,^14^ since their higher polarity caused the greatest surface damage to the NCs and removal of the original long-chain ligands immediately after purification.^13,22,40^ However, we found the opposite trend with the LARP-synthesized NCs (Figure 2(a)), where the acetonitrile-washed NCs were the most stable. We compared with HI-synthesized CsPbI_3_ perovskite NCs prepared using a better-optimized recipe that had a higher baseline stability than the LARP-synthesized NCs. Acetonitrile-treated NCs again exhibited the highest stability (Figure 2(b)), in contrast to expectations based on solvent polarity alone.

**Figure 2:**
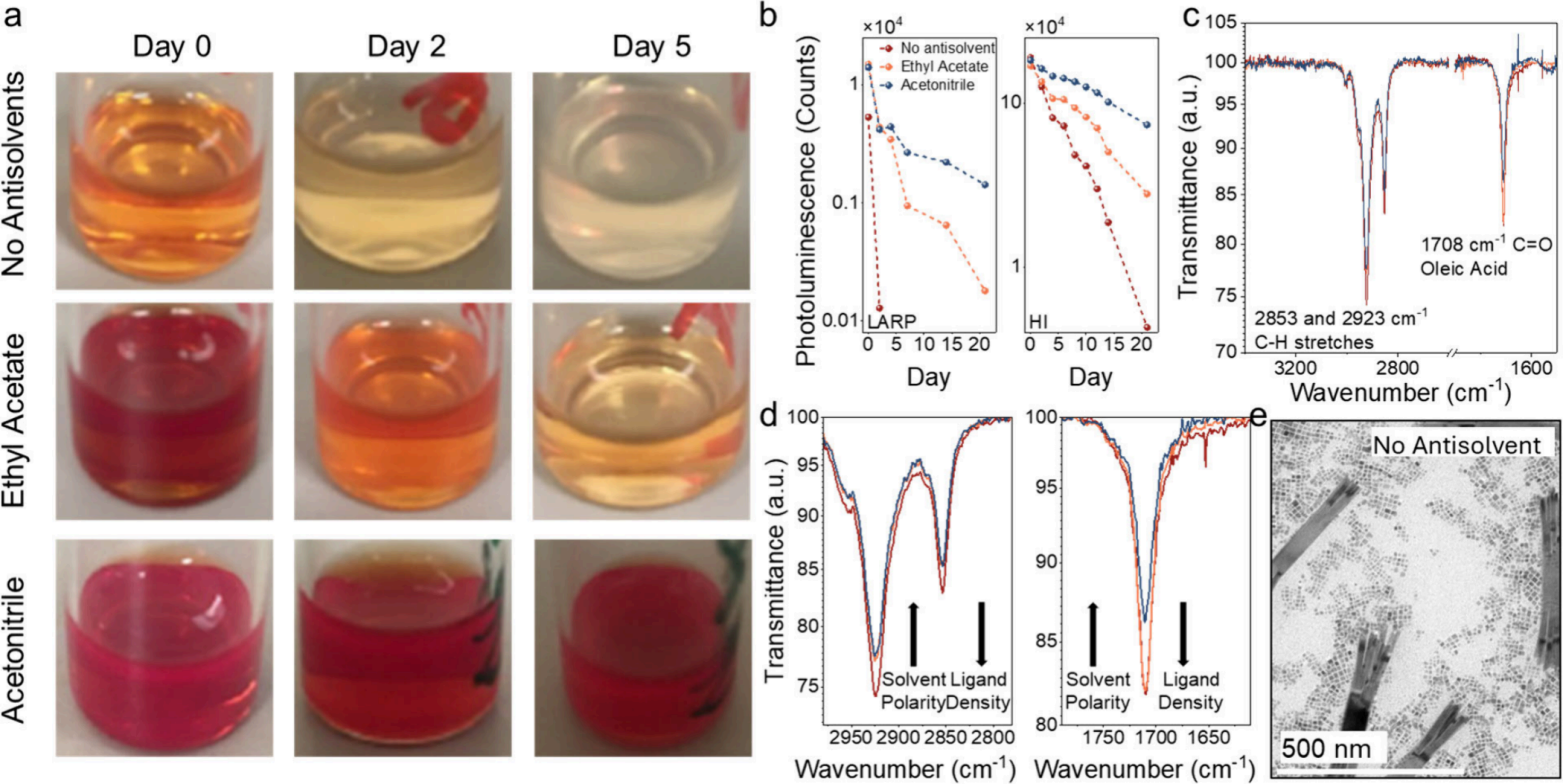
Stability of colloidal CsPbI_3_ NCs purified without antisolvents, and with ethyl acetate and acetonitrile. (a) Photographs of colloidal solutions of LARP-synthesized NCs after storage in ambient air. (b) Change in the PL intensity of LARP- (left) and HI-synthesized NCs (right) in colloidal solution over time in air at room temperature. (c) FTIR spectra of LARP colloidal NC solutions, with key regions of interest zoomed in in part (d). (e) TEM images of LARP NCs purified without antisolvents.

To understand the underlying mechanisms behind these effects, we hereafter focus our detailed characterization on the LARP-synthesized NCs, since the trends between the NCs made using the LARP and HI methods are the same. FTIR measurements confirmed that those purified without antisolvents had the highest density of oleate and oleylammonium ligands (Figure 2(c) and (d)), and acetonitrile-washed NCs the lowest. The rapid degradation of the NCs washed without antisolvents is likely due to residual precursors and impurities inducing the formation of δ-phase structures,^29,41^ which are evident from TEM measurements (Figure 2(e)). These bulk impurities, along with excess surface ligands, disrupt surface equilibria and accelerate degradation.

Given the unexpected improvement in colloidal stability with acetonitrile washing, we hypothesize that these molecules bind to the surface of the perovskite NCs to improve their colloidal stability (Figure 3(a)). That is, we propose that acetonitrile, being the more polar solvent, induces the greater removal of these ligands,^11^ creating more space on the surface for acetonitrile molecules to bind. When the NCs are exposed to air, moisture induces the removal of organic ligands (oleic acid and oleylamine) from the NC surface by distorting the ligand-perovskite surface charge equilibrium, which triggers the detachment of ligands and accelerates degradation (Figure 3(a)). But we found that acetonitrile could be sufficiently strongly bound to the perovskite surface to protect it from degradation (Figure 3(a)). By contrast, ethyl acetate more weakly binds to the perovskite surface, and is mostly removed in the supernatant, thereby not providing any extra protection to the perovskite against moisture-induced degradation.^42,43^

**Figure 3:**
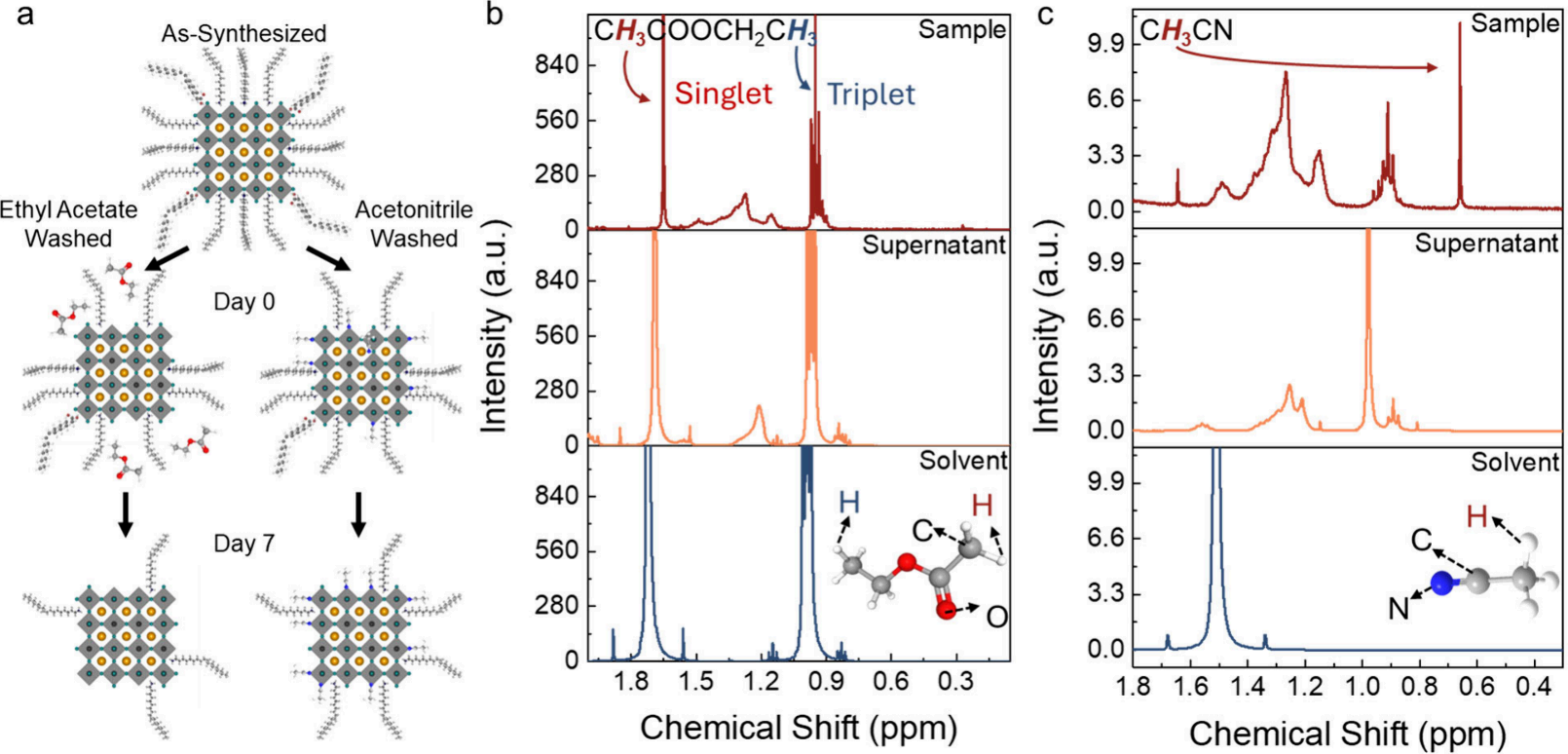
(a) Proposed change in the in NC surface chemistry over time after purification with ethyl acetate versus acetonitrile. The NCs begin by being coordinated with oleate and oleylammonium on the surface, which are then partially removed during purification with polar antisolvents. But when using acetonitrile, these can bind to the perovskite surface by forming Lewis acid:base adducts. ^1^H NMR spectra of the LARP NC sample and supernatant in comparison with that of the pure antisolvent for (b) ethyl acetate and (c) acetonitrile. The solvent panels are the pure antisolvent molecules dispersed in d-toluene. The supernatant panels are the supernatant solutions obtained after the final centrifugation step, and subsequently added to d-toluene. The sample panels are the NCs collected and redispersed in d-toluene after the final centrifugation step.

To test our hypothesis, we first performed ^1^H NMR analysis on the NCs and the supernatants after purification (Figure 3(b) and (c)). In the case of using ethyl acetate, there is no obvious chemical shift in the peaks associated with the hydrogens from the two methyl groups (highlighted in red and blue in Figure 3(b)), as shown in Table 1. This is consistent with little interaction between ethyl acetate and the perovskite surface. By contrast, the peaks associated with hydrogen atoms from the methyl group in acetonitrile (highlighted in red in Figure 3(c)) reduce from around 1.5 ppm (pure acetonitrile, checked from multiple sources of acetonitrile, Figure S8b, SI) to 0.98 ppm (supernatant) to 0.66 ppm (NCs). Fulmer et al. reported that trace impurities of acetonitrile in d-toluene can have a chemical shift at around 0.69 ppm.^44^ This indicates a significant change in the chemical environment for the methyl group in acetonitrile when NCs are present. A reduction in chemical shift can arise from an increase in the electron density around the hydrogen atoms to increase shielding. To rule out other possible assignments of the 0.66 ppm chemical shift, such as water (typically water in d-toluene is at ∼0.43 ppm),^44^ we measured the acetonitrile-washed NCs after 3 days of ambient storage. The peak remains at 0.66 ppm after storage, indicating that acetonitrile molecules persist on the NC surface over time (Figure S8c, SI). Notably, the peak intensity decreases after 3 days of storage in air, suggesting slight NC degradation or ligand detachment, which further excludes water as the origin of this peak at 0.66 ppm, since water-related signals would be expected to increase upon ambient exposure. Furthermore, we only observed the chemical shift at 0.66 ppm for the acetonitrile-treated NC sample. Pure acetonitrile, and the ethyl acetate treated NC sample did not have peaks at 0.66 ppm (Figure S7a, SI), indicating water as an unlikely origin for this peak. Considering the possible interactions between acetonitrile and the perovskite surface, the most likely is between the lone pair of electrons on N from the cyanide group (Lewis base) and undercoordinated Pb^2+^ species on the surface of the perovskite (due to I vacancies), which act as the Lewis-acidic centers.^45,46^ This interaction alters the electronic polarization of the nitrile group, reducing its effective electron-withdrawing character and increasing magnetic shielding of the acetonitrile methyl protons.

**Table 1:** Chemical Shifts from ^1^H NMR Spectra for the Antisolvents, Supernatants, and LARP Colloidal NC Samples (Purified and Redispersed in d-Toluene)^a^

sample type	ethyl acetate (singlet) chemical shift (ppm)	ethyl acetate (triplet) chemical shift (ppm)	acetonitrile chemical shift (ppm)
antisolvent	1.72	0.98	1.51
supernatant	1.69	0.96	0.98
NC sample	1.65	0.95	0.66

a Here, the supernatant is from the solution after the final centrifugation step, and subsequently added to d-toluene. The NCs measured are obtained after the final centrifugation step and redispersed in d-toluene. For ethyl acetate, the hydrogens are labeled based on whether they are associated with the singlet or triplet ^1^H NMR peak (refer to Figure 3b).

To gain a deeper understanding of the surface chemistry of the NCs after purification, we performed XPS measurements on drop-cast NC films, as shown in Figure 4(a) and (b). There is no change in the Pb 4*f* core level peaks from NCs purified without antisolvents and those purified with ethyl acetate, while the acetonitrile-purified sample shows a shift toward lower binding energy. This is consistent with acetonitrile molecules binding to the surface Pb species on the perovskite. The binding energy can be reduced when there is an increase in the density of negatively charged electrons (i.e., increase in Madelung potential) around the Pb^2+^ cation.^47^ Similarly, the N 1*s* core level spectra show no change after washing with ethyl acetate, with a peak centered at 395 eV, associated with the N from the oleylammonium ligands. However, when purified with acetonitrile, there is an additional peak at lower binding energies. This separate peak may be from the cyanide group of acetonitrile bonding to Pb^2+^. The broader fwhm of the oleylammonium-derived N 1*s* XPS core peak originates from enhanced vibronic broadening associated with the NH_3_ functional group, which has multiple vibrational modes and a less rigid chemical environment,^48^ whereas the narrower Pb–N peak reflects a more uniform and well-defined metal–nitrogen bonding state. Furthermore, we directly observed the C≡N signature peak in FTIR spectra (∼2300 cm^–1^)^49^ of the acetonitrile-treated perovskite NC sample, but this peak was absent in the ethyl acetate-treated NC sample (Figure S9, SI). Together, the FTIR and XPS Pb 4*f* and N 1*s* measurements provide experimental support for acetonitrile bonding to Pb surface species in the perovskite NCs.

**Figure 4:**
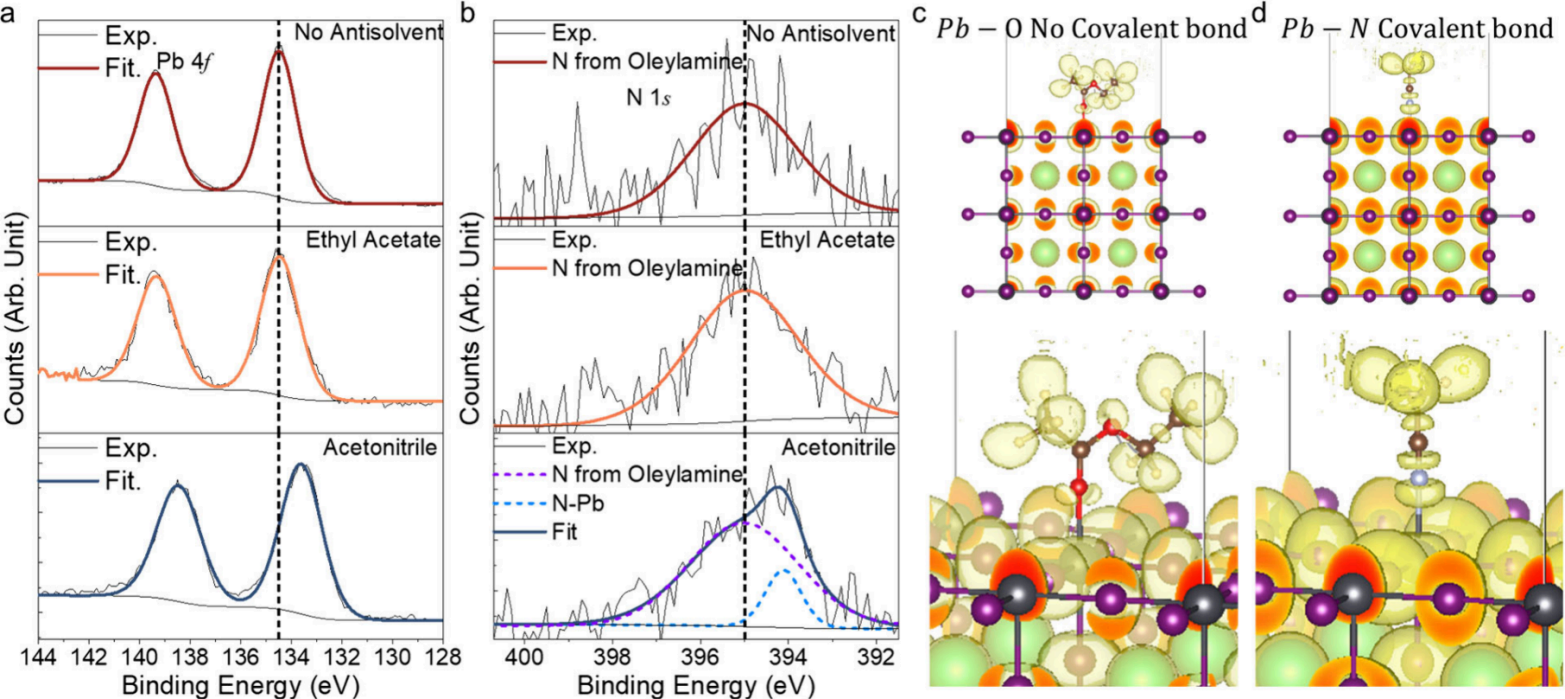
Experimental and computational investigation into Pb–O and Pb–N bonding with ethyl acetate and acetonitrile ligands. (a) Pb 4*f* and (b) N 1*s* core level spectra of LARP NCs purified without using antisolvents, and with the use of ethyl acetate or acetonitrile. Electron Localization Functions^50,51^ calculated for (c) Pb–O bonds between ethyl acetate and the perovskite surface, and (d) Pb–N bonds between acetonitrile and perovskite.

To gain further insights, we conducted density functional theory (DFT) calculations using the electron localization function (ELF).^52^ We found that the calculated electron density (represented by shaded yellow areas) between the O groups in ethyl acetate and Pb do not overlap (Figure 4(c)). The value of the ELF is 0.77, which is below the standard value (0.85) for weak interactions,^53^ suggesting that the lone pairs on O are not donated to Pb^2+^. In contrast, we observe an overlap in the electron densities of N lone pairs and Pb orbitals. The ELF (0.87) exceeds the threshold value of 0.85, indicating the formation of Pb–N covalent bonds. Furthermore, acetonitrile has a larger binding energy (0.15 eV) to the perovskite surface than ethyl acetate (0.12 eV). This is estimated based on the surface energy change before and after antisolvent molecule adsorption to the perovskite surface, where the perovskite surface is fixed as the substrate. A larger binding energy indicates a higher chance of molecule attachment to the NC surface.^54^ These computational results therefore support acetonitrile binding to the perovskite surface.

Finally, to determine the impact of strong Lewis base antisolvents on the performance of treated NCs in devices, we prepared CsPbI_3_ nanocube LEDs following a previously reported procedure.^55^ The device architecture was ITO/PEDOT:PSS/poly-TPD/nanocrystals/TPBi/LiF/Al. We first directly compared devices with NCs that did not have any postligand treatment (Figure S10 and Table S3, SI). Without purification, the density of long-chain organic ligands on the NCs was too high for efficient charge-injection, and the LEDs were nonemissive. LEDs based on acetonitrile-treated NCs exhibited a higher external quantum efficiency (EQE) of 0.86% than those based on ethyl acetate-washed nanocrystals (0.61%), along with a higher maximum luminance and lower turn-on voltage. This improvement is attributed to the more effective removal of long-chain insulating ligands by acetonitrile, resulting in reduced turn-on voltage and enhanced current density after turn-on (Figure S10a,b, SI). To further improve device performance, these purified NCs were subsequently treated with phenethylammonium iodide (PEAI) and triphenylphosphine oxide (TPPO), which are widely used for surface passivation.^55^ Postligand treatment is again more effective for acetonitrile-washed nanocrystals, yielding a peak EQE of 18.1%, compared to 14.9% for ethyl acetate-washed nanocrystals (Figure 5). These results demonstrate that acetonitrile is the more effective antisolvent for nanocrystal purification, despite its higher relative polarity. The more efficient removal of native long-chain ligands, along with more effective surface passivation by acting as a Lewis base to the perovskite surface, enables improved charge injection and more effective post-treatment, ultimately leading to superior device performance. Indeed, the passivation of the perovskite nanocrystal surface by the Lewis base acetonitrile ligands may have played an important role in preventing the agglomeration of the nanocrystals after removing a high density of the original ligands. The resulting devices therefore maintained a color-pure, sharp electroluminescence peak (Figure 5c, inset), with Commission Internationale de l’Éclairage (CIE) coordinates of (0.686, 0.281), very close to the Rec.2020 standard for ultrahigh definition displays.^15^

**Figure 5:**
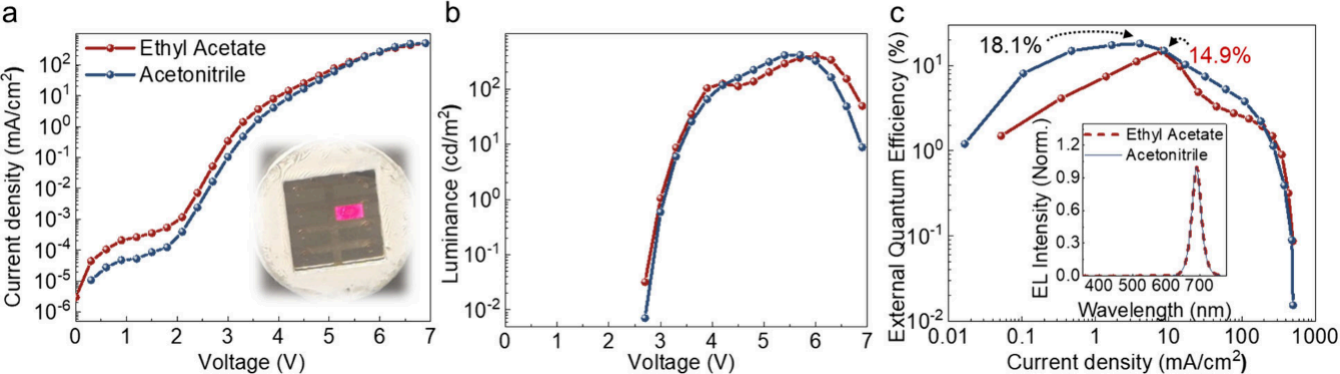
LED device performance for CsPbI_3_ NCs purified using ethyl acetate or acetonitrile, and post-treated with PEAI and TPPO. (a) Current density–voltage (*J*-*V*) curves. (b) Luminance–voltage (*L*-*V*) curves. (c) External quantum efficiency (EQE)–current density curves. Inset in panel (a) is a photograph of the LEDs under operation, while the inset of panel (c) is the normalized electroluminescence spectra of the LEDs made from ethyl acetate or acetonitrile-purified NCs. The CIE coordinates are (0.686,0.281).

In conclusion, we made the surprising finding that purifying α-CsPbI_3_ NCs with the more polar acetonitrile antisolvent led to improved colloidal stability than using the lower polarity ethyl acetate or purifying without using any antisolvent. This is not explained by the current understanding in the literature, where we would have predicted the opposite trend to that which we observed. Through detailed ^1^H NMR, FTIR and XPS measurements, along with DFT calculations, we found that this was due to acetonitrile being able to bind to the surface of the NCs through Pb–N coordination bonds. We showed that Pb–O bonds are not formed with ethyl acetate, explaining why an improvement in stability was not found using this antisolvent. Washing with acetonitrile removes more of the original oleate and oleylammonium ligands, and these gaps in the surface are partly filled with acetonitrile, which helps to protect the perovskite surface from degradation due to moisture-induced ligand removal. This work shows that designing more effective antisolvents requires consideration of how the antisolvents interact with the NC surface, and not solely how they influence the ligands bound to the surface. Using moderately polar antisolvents is advantageous for more easily separating NCs from impurities. Importantly, we demonstrate that the stabilization imparted by antisolvent choice extends beyond surface chemistry effects, and yields measurable improvements in optoelectronic device performance. Our work shows that selecting antisolvents that act as strong Lewis bases, such that they can passivate the perovskite surface, enable high colloidal stability and optoelectronic properties, which are highly desirable for optoelectronic applications.

## Supplementary Material



## Data Availability

Raw data for the main text and Supporting Information available from the Oxford Research Archive repository from the following link: https://doi.org/10.5287/ora-mnqx84mjp with the DOI: 10.5287/ora-mnqx84mjp.

## Notes

Experimental details; Data mining of the antisolvents used for perovskite nanocrystal purification; Additional XRD, PL, NMR, and UV–vis spectra; Additional references (PDF)
