# Supplementary material for: Stronger Lewis Base Antisolvents Improve Perovskite Nanocrystal Stability
Source: ACS Energy Lett. 2026 Apr 9;11(5):3993–4001. doi: 10.1021/acsenergylett.6c00480 (PMC13162302; doi:10.1021/acsenergylett.6c00480)
Supplement: Supplementary file 1 [file nz6c00480_si_001.pdf]

Supporting Information for

Stronger Lewis Base Antisolvents Improve Perovskite Nanocrystal Stability

Junzhi Ye [a,b#], Charlie Nicholls [a#], Woo Hyeon Jeong [a], Dong Yoon Chung [a], Ashish Gaurav [a], Kieran De-Ville [a], Rui Xu [c], Zongming Ni [d], Qingyu Wang [e], Xinyu Shen[e], Jieling Tan [f], Eilidh L. Quinn [a], Maxime Atkinson [a], Wei Zhang [f], Haitao Zhao [g], Henry J. Snaith [e], Robert A. Taylor [e], Yunwei Zhang [c], Robert L. Z. Hoye [a]**

[a] Department of Chemistry, University of Oxford, Inorganic Chemistry Laboratory, Oxford, OX1 3QR, United Kingdom

[b] Institute of Polymer Optoelectronic Materials and Devices, Guangdong Basic Research Centre of Excellence for Energy & Information Polymer Materials, State Key Laboratory of Luminescent Materials and Devices, School of Materials Science and Engineering, South China University of Technology, Guangzhou 510640, China

[c] School of Physics, Sun Yat-sen University, Guangzhou, 510275, China

[d] Wenzhou Institute of Technology, Wenzhou, 325000, China

[e] Clarendon Laboratory, Department of Physics, University of Oxford, Oxford, OX1 3PU, United Kingdom

[f] Center for Alloy Innovation and Design (CAID), State Key Laboratory for Mechanical Behavior of Materials, Xi'an Jiaotong University, Xi'an, 710049, China

[g] Research Centre for Materials Intelligent Manufacturing, State Key Laboratory of Ultra-precision Machining Technology, Department of Electrical and Electronic Engineering, The Hong Kong Polytechnic University, Hong Kong, China

[#]J.Y. and C.N. contributed equally to this paper.

Corresponding Authors

*Junzhi Ye, Email: junzhiye1994@scut.edu.cn

*Robert L. Z. Hoye, Email: robert.hoye@chem.ox.ac.uk

Methods

Data Mining:

The literature data were collected from various journals and publishers, including ACS, Elsevier, Frontiers, MDPI, Nature, RSC, Science, and Wiley, focusing on studies related to nanoplatelet materials. This dataset includes information on perovskite nanocrystals. To extract details on synthesis methods and purification solvents, we employed the open-source DeepSeek model.[1] Given the complexity and variability of natural language, we opted against direct keyword-based extraction and instead embedded key terms within a well-structured contextual framework to enhance accuracy.[2] To ensure clarity and consistency in the extracted information, a standardized output template was implemented, facilitating subsequent data processing. The extracted data were then refined and normalized through coding, ensuring uniformity and scientific rigor in statistical analysis (e.g., standardizing terms such as "pure water" and "H_2O" to "water"). Finally, based on the processed data, we generated statistical visualization of the types of antisolvents used, and their frequency of use, both with and without consideration of synthesis methods. The results are shown in Figure S1.

Synthesis:

Materials: Lead (II) Iodide (PbI_2, 99%, Sigma Aldrich), oleylamine ($C_{18}H_{37}N$, 70%, Sigma Aldrich), oleic acid ($C_{18}H_{34}O_2$, 90%, Sigma Aldrich), cesium carbonate (Cs_2CO_3, ≥98.00%, TCI) (cesium acetate (CH_3COOCs, ≥99.99%, Sigma Aldrich), ethyl acetate for HPLC ($CH_3COOC_2H_5$, ≥99.7%, Sigma Aldrich), toluene ($C_6H_5CH_3$, 99.8%, Sigma Aldrich), hexane (C_6H_{14}, ≥95%, Sigma Aldrich), acetonitrile (CH_3CN, >99.9%, ROMIL), phenethylammonium iodide (PEAI) (GreatCellSolar), chlorobenzene (anhydrous 99.8%, Sigma Aldrich), triphenylphosphine oxide (TPPO, 98%, Sigma Aldrich), poly[bis(4-phenyl) (4-butylphenyl) amine] (poly-TPD, Sigma Aldrich) and 2,2′,2″-(1,3,5-benzinetriyl)tris(1phenyl-1-H-benzimidazole) (TPBi; 99.9%, 1-materials) and deuterated toluene (>99, toluene-d_8 from Sigma Aldrich).

LARP-based Synthesis:

Preparation of lead iodide solution: 46.1 mg (0.1 mmol) of PbI_2 was added to 10 mL toluene in ambient air with 100 µL of both OAm and OA, and heated at 60 °C with continuous stirring until completely dissolved.

Preparation of Cs(oleate): 38.9 mg (0.2 mmol) of Cs(acetate) was dissolved in 4 mL OA in ambient air, and heated at 60 °C with continuous stirring until completely dissolved.

Nanocrystal synthesis: 2 mL of PbI_2 solution was continuously stirred at a calibrated temperature at 80 °C for 10 min in ambient air, inside a fume hood. 150 µL of Cs(oleate) was added and allowed to react for 10 s. The solution was then centrifuged at 3000 rpm (875 *g*) for 3 min using a LISA 2.5 L Centrifuge. The resulting supernatant was transferred to a separate centrifuge tube and a 0.3 to 1 ml of antisolvent was added before being centrifuged at 12000 rpm (14000 *g*) for 5 minutes. The supernatant was then discarded and the resulting solid redispersed in 1 mL hexane before being filtered with a PTFE 0.2 µm filter. We normally waited for 30 minutes in between two sample preparations to avoid the centrifuge accumulating excess heat when spinning at high speed.

HI-based Synthesis:

Preparation of Cs(oleate):

100 mg (0.307 mmol) of Cs_2CO_3 was carefully weighed and added to a 100 ml three-neck flask along with 1 mL of OA (oleic acid) and 10 mL of ODE (octadecene). The flask was kept under vacuum for 40 minutes at 100 °C. Then, the temperature of the solution was maintained at 100 °C under an argon flow. The solution was continuously heated and stirred until it turned transparent.

Nanocrystal synthesis: For the PbI_2 solution, 173 mg (0.375 mmol) of PbI_2, 10 mL of ODE, 1 mL of OA, and 1 mL of OAm were added to a 100 mL three-neck flask. The flask was stirred at 90 °C and 800 rpm under vacuum to remove residual gases and impurities. After 30 minutes, the temperature was increased to 150 °C while purging the flask with argon gas, which was then maintained at a constant flow. Once the temperature reached 150 °C, 0.8 mL of Cs-oleate was rapidly injected into the flask. Three seconds after the injection, the reaction mixture was quenched by immersion in an ice bath to achieve rapid cooling. For purification, the prepared NCs were transferred to a 50 mL centrifuge tube, and the desired amount of antisolvent (such as ethyl acetate or acetonitrile) was added. The mixture was centrifuged at 8000 rpm (6225 *g*) for 5 minutes at 24 °C. The supernatant, containing impurities and excess solvent, was carefully decanted, leaving the NCs as a precipitate at the bottom of the tube. A fixed amount of hexane was then added, followed by centrifugation at 6000 rpm (3502 *g*) for 5 minutes. After centrifugation, the supernatant containing the purified NCs dispersed in hexane was collected.

Optical characterization:

Ultraviolet-Visible Spectrophotometry (UV-Vis): A Shimadzu UV-2600 spectrometer was used, and measurements were performed on colloidal solutions over a 400-700 nm range using 1 mm thick quartz cuvette from Hellma®. Samples were redispersed in hexane and then dissolved in addition hexane in the cuvette.

Photoluminescence (PL) Measurements and Photoluminescence Quantum Yield (PLQY): Photoluminescence spectra of NC solutions were obtained using a 405 nm wavelength, 1 mW power laser linked to an Ocean Insight High Performance Spectrometer. The PLQY measurements were carried out using the same 405 nm 1 mW laser with an 80 mm integrating sphere linked to an Ocean Insight High Performance Spectrometer. All samples were measured using a quartz cuvette in air.

Time resolved photoluminescence lifetime (TRPL) measurements: The laser used in this work is a Coherent Mira 900 Ti:Sapphire laser fitted with a pulse picker. An Inrad 5-050

ultrafast harmonic generation system is used for the generation of the 400 nm wavelength pump beam (second harmonic). The second harmonic is used as the excitation source for PL. The repetition rate is 7.6 MHz and the beam used had a 1 μm diameter. The laser is focused through a confocal microscope onto the samples, where the reflected PL is passed back through the microscope objective into a Princeton Instruments 0.3 m spectrometer and Picoquant PMT connected to a Timeharp Picoquant time-correlated single photon counting system. All samples were prepared by drop casting the solutions onto thin silicon wafers for analysis. The excitation source had a pulse width of 120 fs.

Structural and surface chemistry characterisation:

X-Ray Diffraction (XRD): X-Ray diffraction patterns of the $CsPbI_3$ NCs were carried out using an X-Ray Diffractometer (Bruker D8 Advance powder X-ray diffractometer) equipped with a Cu-K_α X-ray tube. Films were produced by drop casting 200 μL of solution onto a 3 mm circular glass slide and placed in an air-free sample holder.

^{1}H NMR Proton Nuclear Magnetic Resonance (^{1}H-NMR): Liquid-phase ^{1}H-NMR measurements were taken at room temperature using a Bruker Avance III HD nanobay NMR equipped with a 9.4 T magnet at 400.2 MHz. Toluene-d_8 was employed as the solvent and chemical shifts were referenced using the CH_3 trace at 2.09 ppm. All of the samples were prepared using 400 μL of toluene-d_8 with the addition of 300 μL of solution. Solutions of PbI_2 were prepared, as usual, using toluene-d_8. Toluene-d_8 was used instead of hexane to redisperse the solid NC samples. Residual non-deuterated toluene present in toluene-d_8 served as an internal standard for quantifying the native ligand concentration of the NCs after washing.

Fourier Transform Infrared Spectroscopy (FTIR): Fourier Transform Infrared Spectroscopy (FTIR) was measured using Transmission IR spectroscopy (Shimadzu Affinite-1). Liquid phase FTIR was used to measure the infrared spectra of NC sample and supernatant solutions. Samples were prepared using the synthesis in 3.1 with 200 μL of solution drop casted onto the spectrometer. The range of the spectrometer was from 3600 cm^{-1} to 600 cm^{-1}.

Transmission Electron Microscopy (TEM): Samples were prepared by putting a small drop of nanoparticle solution onto a carbon-coated copper grid (Sigma Aldrich) in a glovebox. TEM images were recorded using a Talos L120C G2 operated at 120 kV.

X-ray photoemission spectroscopy: XPS data was acquired using a Kratos Axis SUPRA using monochromated Al K_α (1486.69eV) X-rays at 12 mA emission and 15 kV HT (180 W), with an analysis area of 700 × 300 μm^2. All data was recorded at a base pressure of below 9×10^{-9} Torr and at room temperature (294 K). The results were analysed using the software CasaXPS v2.3.19PR1.0. The peak fitting was done using a built-in Shirley background function and GL (Gaussian/Lorentzian Product Form) function in CasaXPS. The selection of the fitting function is based on the least residual after fitting.

Density Functional Theory Calculations:

The DFT calculations employed a 2 × 2 × 1 slab model as the substrate for surface adsorption

studies. The DFT calculations were conducted using density functional theory within the generalized gradient approximation with the Perdew−Burke−Ernzerhof (PBE) functional[3] for the exchange−correlation energy, as implemented in the Vienna Ab Initio Simulation Package (VASP) code.[4] The plane-wave kinetic energy cutoff of 600 eV for the plane-wave expansion and Monkhorst-Pack k-point mesh resolution of 13 × 13 × 1 in reciprocal space for all of the structures in the Brillouin zone were chosen to ensure an energy convergence and residual forces within 1 meV/atom and 1 meV/Å, respectively. The electron localization function (ELF) was calculated and plotted to explore the bonding mechanism of surface adsorption.

Device Fabrication:

For $CsPbI_3$-NC LED devices, the ITO substrate was washed with DI water, acetone, and isopropyl alcohol for 10 min each with ultrasonication. After drying, the ITO substrates were treated with UV-Ozone for 20 min. The aqueous PEDOT:PSS solution was spin-coated on ITO at 4500 rpm for 40 s and heat treated at 140 °C on the hotplate for 10 min. Afterwards, poly-TPD (10 mg mL^{-1} in chlorobenzene) was spin-coated at 4000 rpm for 40 and annealed at 120 °C for 5 min in a N_2-filled glove box. The NC solution was subsequently washed with ethyl acetate or acetonitrile, then re-dispersed in octane 30 mg mL^{-1}. This colloidal solution was spin-coated at 2000 rpm for 20 s on the device structures, and this is the device data shown in Figure S10. We also treated with each ligand solution (PEAI and PEAI-TPPO) for 10 s under ambient conditions with 20–30% relative humidity to get better device results, and this is shown in Figure 5. Finally, TPBi (40 nm), LiF (1 nm), and Al (100 nm) were sequentially deposited at $\approx 10^{-6}$ Torr by thermal evaporation. The area of the Al electrode defines a 0.045 cm^2 emission area of the device. The device was measured in an integrating sphere inside a N_2-filled glovebox without encapsulation.

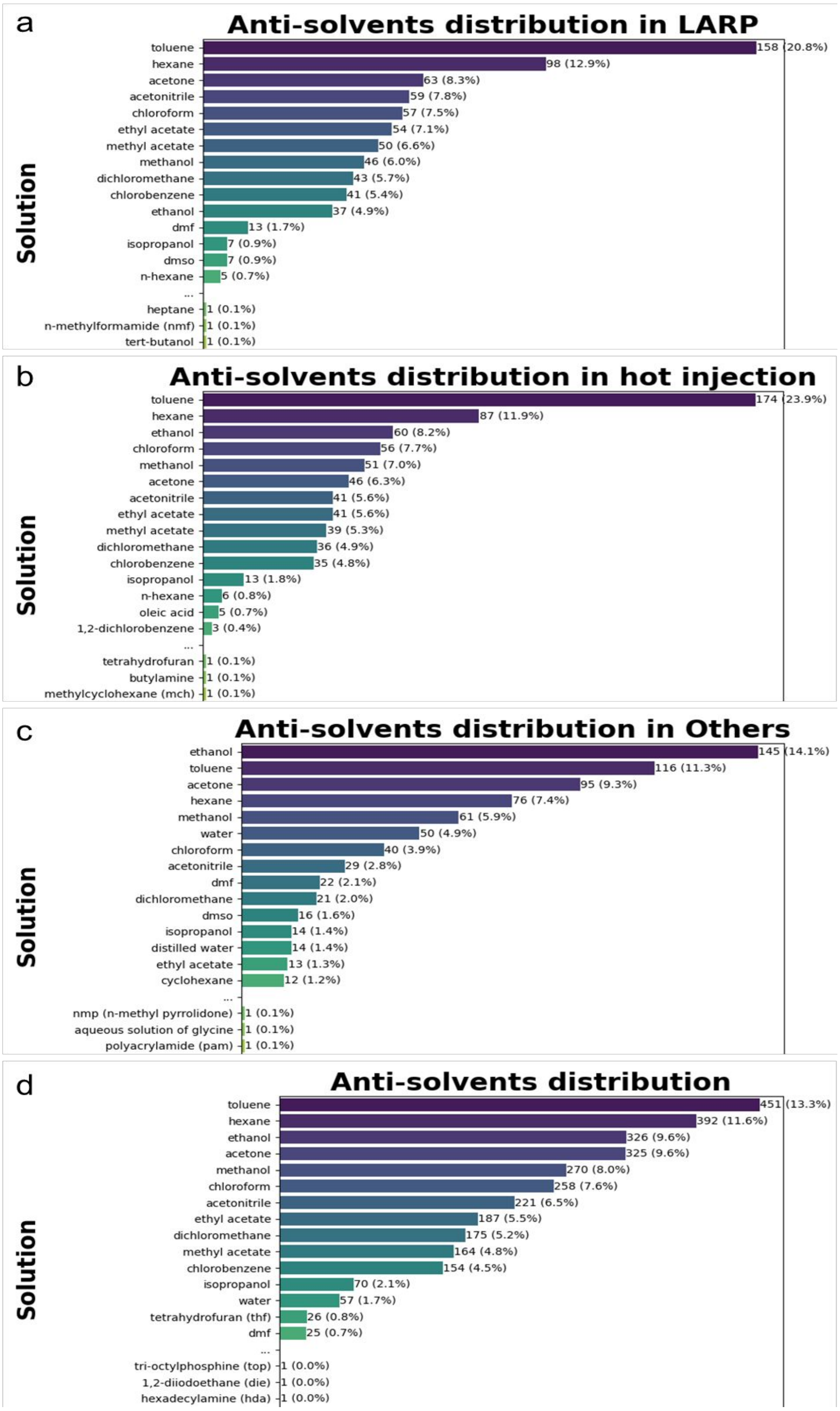

Figure S1. Frequency of reported antisolvents used for nanocrystal purification from data mining through literature. (a) Antisolvents used for ligand-assisted reprecipitation synthesis (LARP). (b) Antisolvents used for hot injection synthesis. (c) Antisolvents used for other synthesis methods. (d) Total antisolvents frequency distribution for different types of synthesis methods.

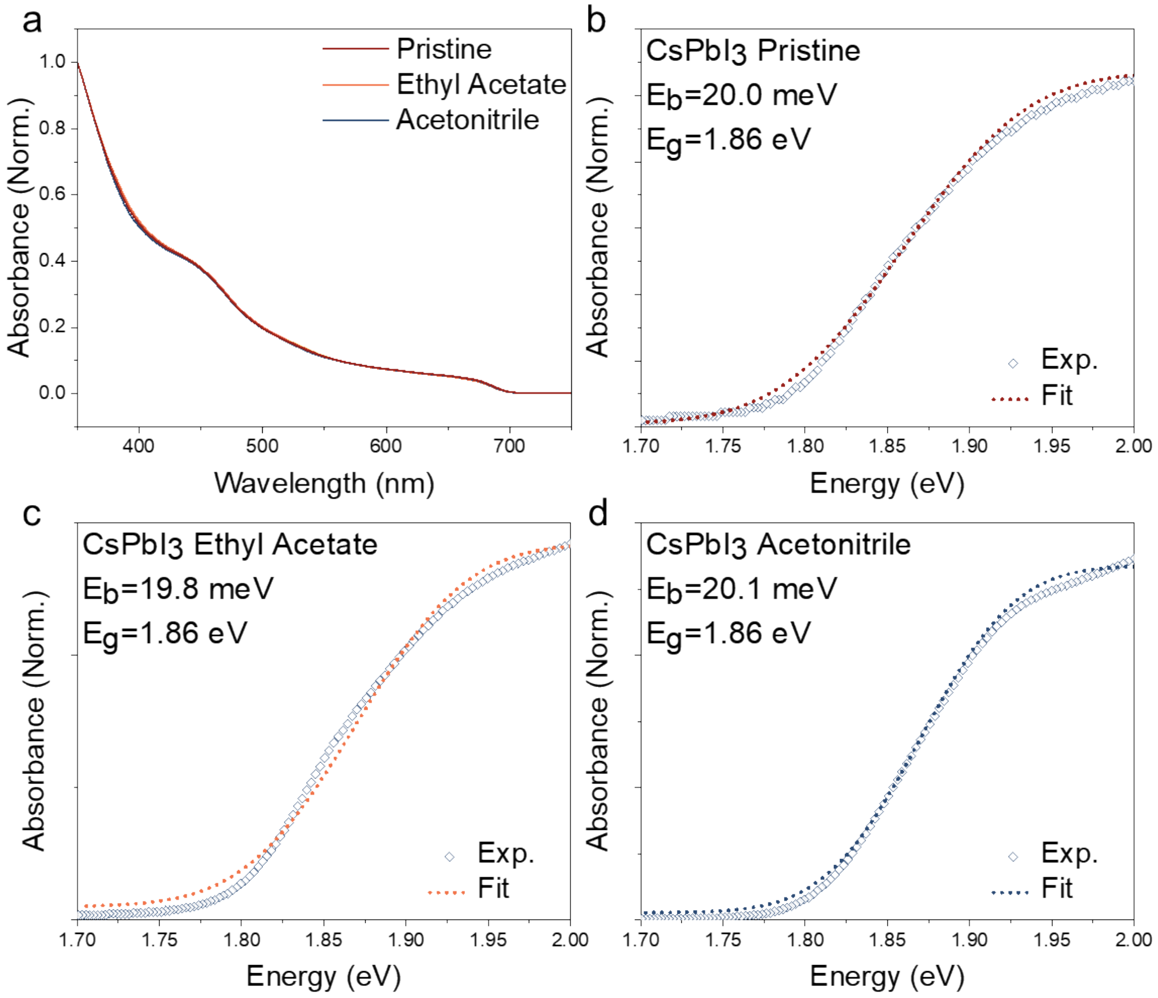

Figure S2. (a) Normalized steady state absorption spectra to confirm relative similar optical density and NC concentration. Elliott model fitting of $CsPbI_3$ NCs purified with (b) no antisolvents, (c) ethyl acetate and (d) acetonitrile. The model fit is based on Ref. 5.

Table S1. Yields of NCs checked by weighing of the centrifuge when it is empty and after collecting the dry precipitates The yields are compared based on 2 mL of PbI_2 precursor solution (0.1 mmol) with 150 μL Cs-precursor solution (0.2 mmol) using LARP method.

Sample	Crude yield / mg
No antisolvent	13.4 ± 0.2
Ethyl Acetate	12.4 ± 0.5
Acetonitrile	14.7 ± 0.1

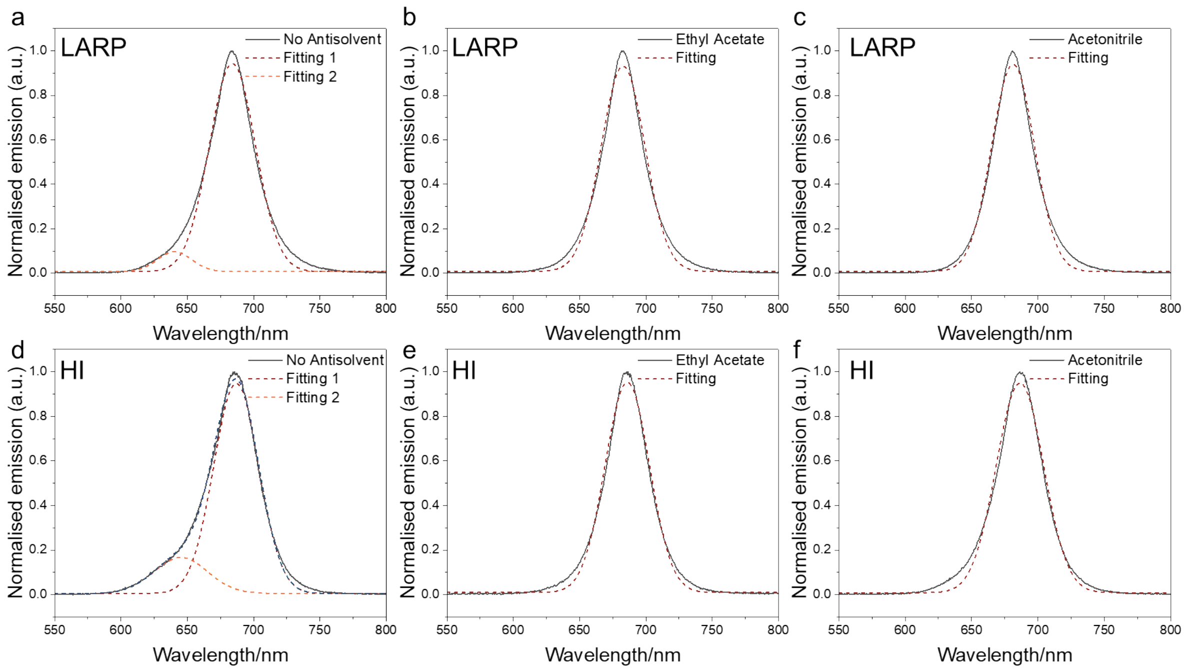

Figure S3. Fitted PL spectra of colloidal $CsPbI_3$ NCs prepared by LARP and purified with (a) no antisolvents, (b) ethyl acetate and (c) acetonitrile. Fitted PL spectra of colloidal $CsPbI_3$ NCs prepared by HI and purified with (d) no antisolvents, (e) ethyl acetate and (f) acetonitrile.

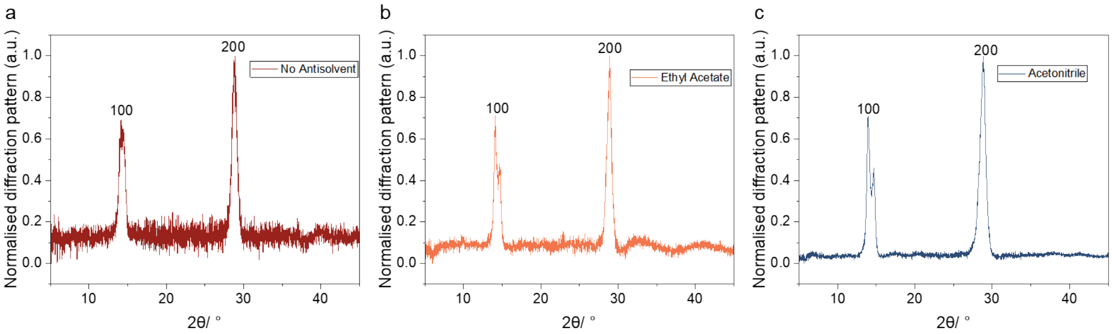

Figure S4. Thin film XRD for LARP-synthesized NCs purified with (a) no antisolvents, (b) ethyl acetate and (c) acetonitrile. The samples were prepared by drop casting NC solution on glass subtrates. The double peak shown in the (100) peak is due to the formation of face-down nanoplatelet superlattices.[6] The superlattice diffraction signal (double peaks) was more obvious in the antisolvent-treated sample due to a narrower size distribution with more ordered packing.

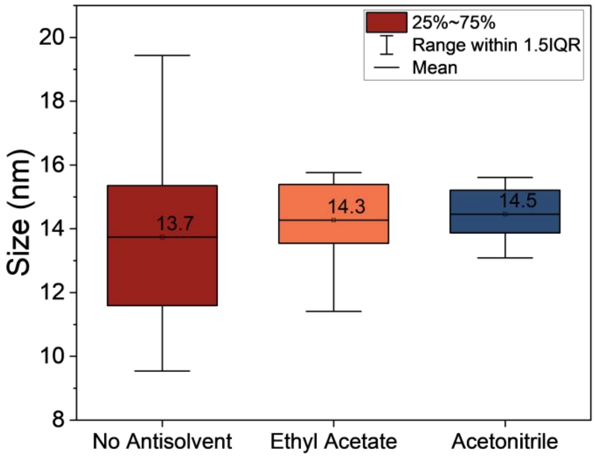

Figure S5. LARP-NC size distribution box plots measured from TEM images shown in Figure 1 in the main text. Each box plot is comprised of 20 data points.

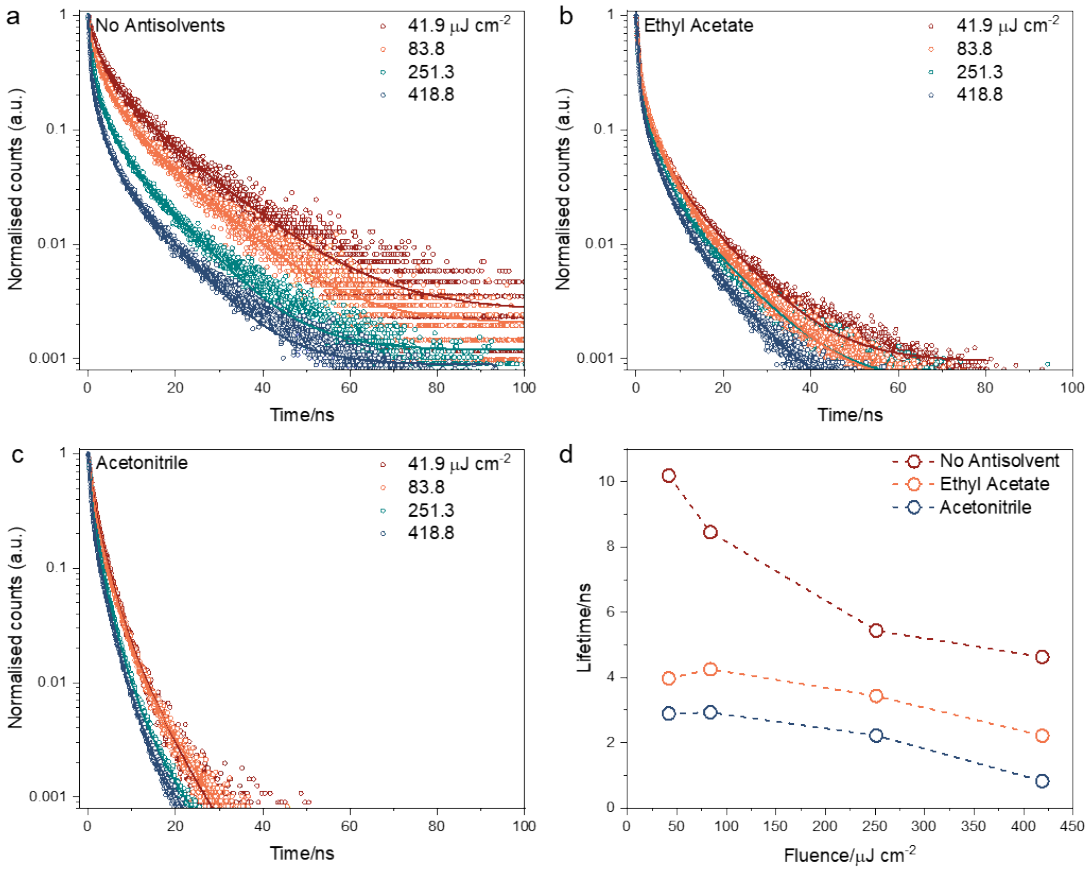

Figure S6. Fluence-dependent PL decay measured using a confocal PL microscope for LARP-NCs purified with (a) no antisolvents, (b) ethyl acetate and (c) acetonitrile. (d) Fitted fluence-dependent average PL lifetime. Excitation was with a 400 nm wavelength pulsed pump laser.

Table S2. Phenomenological triexponential fitting of time-resolved photoluminescence decay measurements of LARP-NCs at 418.8 μJ cm^{-2} excitation fluence. Data in Figure S6.

Sample	A_1	S.D.	τ_1	S.D.	A_2	S.D.	τ_2	S.D.	A_3	S.D.	τ_3	S.D.	τ_{ave}
No Anti	0.697	0.002	0.284	0.001	0.259	0.001	1.870	0.018	0.095	0.001	7.647	0.067	4.63
EA	0.733	0.001	0.196	0.000	0.210	0.001	1.134	0.010	0.058	0.001	4.366	0.044	2.22
ACN	2.383	0.002	0.215	0.001	0.483	0.003	0.870	0.009	0.091	0.002	2.254	0.032	0.82

S.D. is the standard deviation from the fitting.

τ_{ave} is calculated based on the following equation[7]:

$$\tau_{ave} = \frac{A_1\tau_1^2+A_2\tau_2^2+A_3\tau_3^2}{A_1\tau_1+A_2\tau_2+A_3\tau_3} \qquad \text{(Eq. 1)}$$

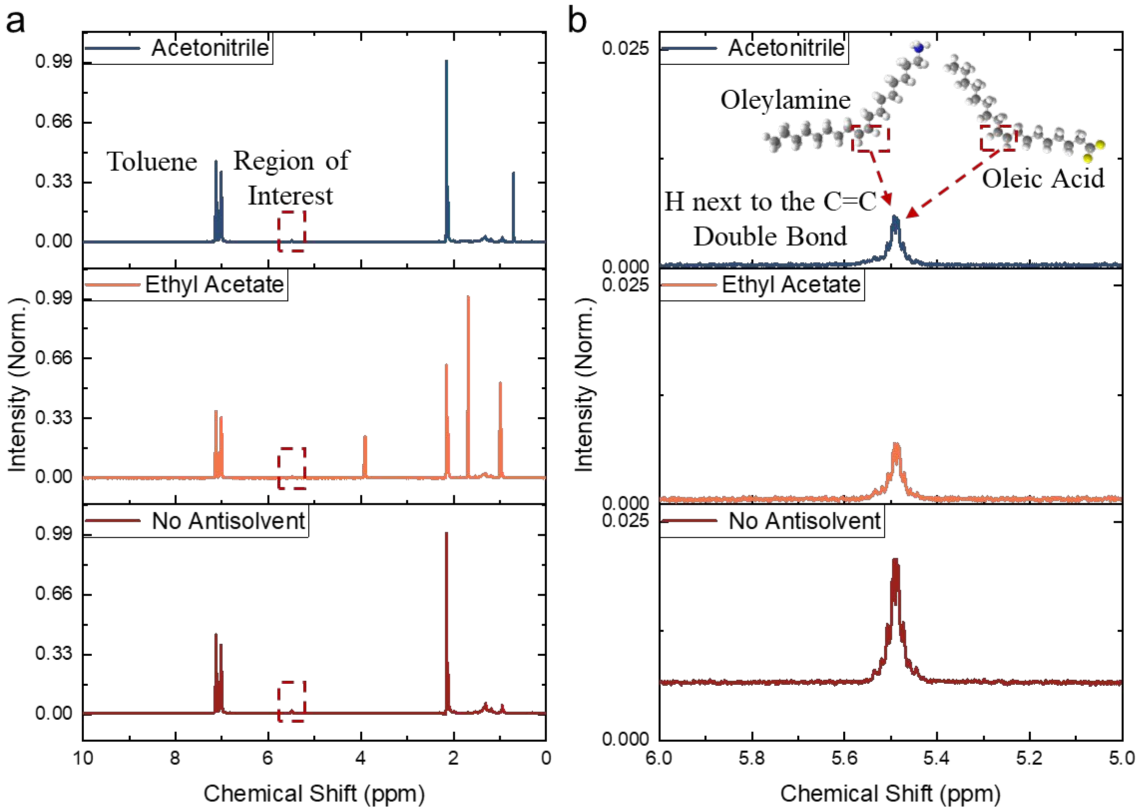

Figure S7. ^{1}H-NMR spectra for NCs in deuterated toluene (toluene-d_8). (a) Full ^{1}H-NMR spectra of NCs treated by acetonitrile, ethyl acetate and no antisolvents. (b) Zoomed-in spectra at 5-6 ppm to show hydrogen signal around C=C in oleic acid and oleylamine ligands.

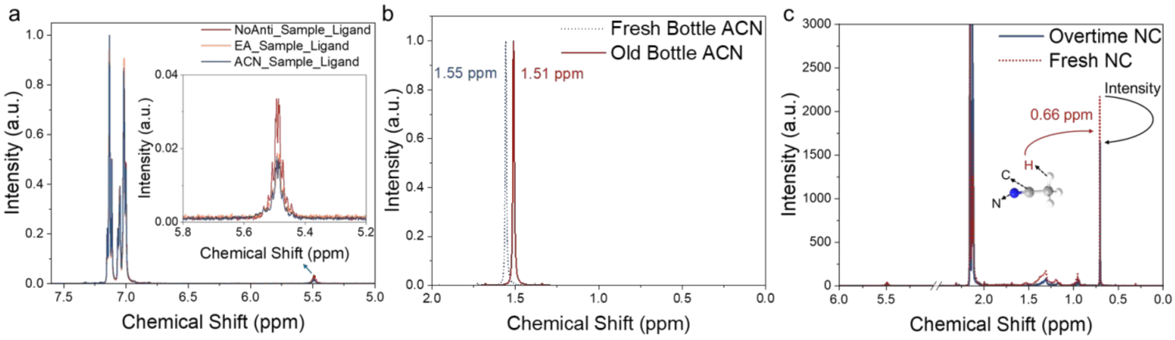

Figure S8. Analysis of ^{1}H-NMR spectra. (a) ^{1}H-NMR spectra normalized to the internal standard of the residual toluene peak at 7.09 ppm in d-toluene. (b) ^{1}H-NMR of perovskite NCs washed with acetonitrile from different sources. (c) ^{1}H-NMR of fresh acetonitrile-washed $CsPbI_3$ perovskite NCs, compared to these NCs stored for 3 days in air.

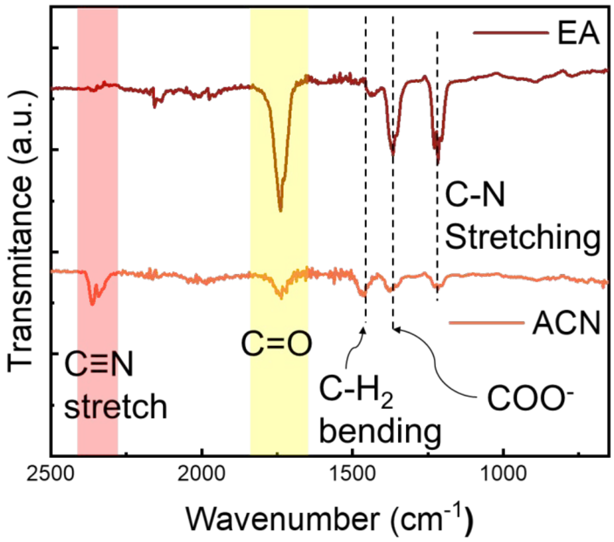

Figure S9. FTIR spectra of ethyl acetate and acetonitrile treated NC thin films. The region in red highlights the C≡N nitrile signature peak in FTIR spectra[8].

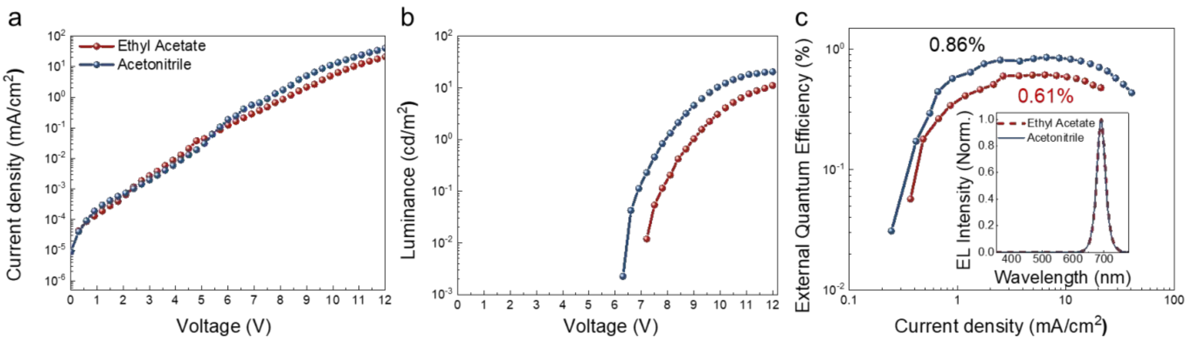

Figure S10. LED device performance for NCs after direct washing with ethyl acetate and acetonitrile without post treatment. (a) Current density-voltage (*J*-*V*) curve. (b) Luminance-voltage (*L*-*V*) curve. (c) External quantum efficiency (EQE)-current density curve. Inset are the normalized electroluminescence spectra of the LEDs.

Table S3. LED device performance before and after ligand treatment of the ethyl acetate (EA) and acetonitrile (ACN) washed NCs.

	Max EQE_{EL} [%] @ bias	Max luminance [cd m^{-2}] @ bias	Turn-on Voltage [V] @ 0.1cd/m^2
	After Ligand Treatment		
EA-NC	14.9@3.9	401@6	3.0
ACN-NC	18.1@3.9	414@5.7	3.0
	Before Ligand Treatment		
EA-NC	0.61@10.2	11.1@12	7.8
ACN-NC	0.86@9.3	20.4@12	6.9

References for Supporting Information

(1) Bevara, R. V. K.; Mannuru, N. R.; Lund, B. D.; Karedla, S. P.; Mannuru, A. Beyond ChatGPT: How DeepSeek R1 may transform academia and libraries? *Library Hi Tech News* **2025**, *42*, 4 - 8. DOI: 10.1108/LHTN-01-2025-0024
(2) Patel, K.; Caragea, C. Exploring Word Embeddings in CRF-based Keyphrase Extraction from Research Papers. In Proceedings of the 10th International Conference on Knowledge Capture, Marina Del Rey, CA, USA; 2019.
(3) Perdew, J. P.; Burke, K.; Ernzerhof, M. Generalized Gradient Approximation Made Simple. *Phys. Rev. Lett.* **1996**, *77* (18), 3865-3868. DOI: 10.1103/PhysRevLett.77.3865.
(4) Kresse, G.; Furthmüller, J. Efficient iterative schemes for ab initio total-energy calculations using a plane-wave basis set. *Phys. Rev. B* **1996**, *54* (16), 11169-11186. DOI: 10.1103/PhysRevB.54.11169.
(5) Naeem, A.; Masia, F.; Christodoulou, S.; Moreels, I.; Borri, P.; Langbein, W. Giant exciton oscillator strength and radiatively limited dephasing in two-dimensional platelets. *Phys. Rev. B* **2015**, *91* (12), 121302. DOI: 10.1103/PhysRevB.91.121302.
(6) Toso, S.; Baranov, D.; Filippi, U.; Giannini, C.; Manna, L. Collective Diffraction Effects in Perovskite Nanocrystal Superlattices. *Acc. Chem. Res.* **2023**, *56* (1), 66-76. DOI: 10.1021/acs.accounts.2c00613.
(7) Ye, J.; Li, Z.; Kubicki, D. J.; Zhang, Y.; Dai, L.; Otero-Martínez, C.; Reus, M. A.; Arul, R.; Dudipala, K. R.; Andaji-Garmaroudi, Z.; et al. Elucidating the Role of Antisolvents on the Surface Chemistry and Optoelectronic Properties of $CsPbBr_xI_{3-x}$ Perovskite Nanocrystals. *J. Am. Chem. Soc.* **2022**, *144* (27), 12102-12115. DOI: 10.1021/jacs.2c02631.
(8) Dereka, B.; Lewis, N. H. C.; Keim, J. H.; Snyder, S. A.; Tokmakoff, A. Characterization of Acetonitrile Isotopologues as Vibrational Probes of Electrolytes. *The Journal of Physical Chemistry B* **2022**, *126* (1), 278-291. DOI: 10.1021/acs.jpcb.1c09572.
